# Facial redness in Japanese adolescents with atopic dermatitis treated with dupilumab: A case series

**DOI:** 10.1016/j.jacig.2023.100096

**Published:** 2023-03-21

**Authors:** Shuhei Hara, Takaaki Itonaga, Makoto Nishino, Noriyuki Yanagida, Sakura Sato, Motohiro Ebisawa

**Affiliations:** a Department of Pediatrics and, Clinical Research Center for Allergy and Rheumatology, National Hospital Organization Sagamihara National Hospital, Sagamihara, Japan; b Department of Allergy, Clinical Research Center for Allergy and Rheumatology, National Hospital Organization Sagamihara National Hospital, Sagamihara, Japan

**Keywords:** Atopic dermatitis, dupilumab, Japanese adolescent, erythema

## Abstract

This case series study is the first report of Japanese adolescents who experienced dupilumab facial redness after starting dupilumab treatment for refractory atopic dermatitis. In every case, dupilumab facial redness subsided without discontinuation of dupilumab within 3 months after onset.

Dupilumab, a human mAb against the IL-4/13 receptor, is the first biologic approved for the treatment of moderate-to-severe atopic dermatitis (AD). In Japan, dupilumab was approved in April 2018 for use in patients aged 15 years or older with refractory AD. The most common adverse reactions reported in clinical trials^1^, ^2^ were injection site reactions and conjunctivitis. However, since dupilumab was first used in daily medical practice, erythema of the head and neck (dupilumab facial redness [DFR]) began to be reported worldwide. A recent systematic review reported more than 100 cases of DFR in adults, adolescents, and children.^3^ In Japan, DFR has been reported in adults^4^ but not in adolescents aged 15 to17 years. This study aimed to investigate the incidence and clinical course of DFR in Japanese adolescents undergoing dupilumab treatment for refractory AD in our hospital. We retrospectively reviewed the medical records of adolescent patients who started receiving dupilumab for refractory AD at our hospital from January 2019 to December 2021. The treatment course was then observed until April 2022. This study was approved by the ethics committee of Sagamihara National Hospital (approval no. 20180401). Written informed consent was obtained from the patients for the publication of photographs. The study information was disclosed on the hospital’s website, thus allowing the study participants the choice to participate.

A total of 13 adolescents treated with dupilumab were identified; all were males, with a median age of 15 years and an Eczema Area and Severity Index (EASI) score of 19.6. During a mean treatment period of 14 months (range 3-33 months), 3 patients had confirmed DFR (Table I). Details of the clinical course of the 3 cases of DFR are described in the following sections.

**Table I:** Patient characteristics

Case	Sex	Age (y)	Weight (kg)	AD onset	EASI score	Previous treatment (face and neck/other parts)	Eos (/μL)	LDH level (IU/L)	Total IgE level (kU/L)	TARC level (pg/mL)	IL-4 level (pg/mL)	Starting dosage of dupilumab	Onset of DFR (days after starting dupilumab)	Any attempt to treat DFR	Complete resolution of DFR after the onset (d)
1	M	16	69.4	Infancy	16.1	TCI /TCS (class Ⅱ)	360	225	1530	389	<2	600 mg (followed by 300 mg given every other week)	73	TCS combined with ketoconazole	116
2	M	15	67.4	Infancy	10.5	TCS (class Ⅱ) /TCS (class Ⅱ)	230	227	12900	1545	7.2	600 mg (followed by 300 mg given every other week)	63	Instructed to apply adequate TCS and undergo observation	105
3	M	17	45	Infancy	25.9	Delgocitinib/Delgocitinib	NA	NA	NA	NA	NA	600 mg (followed by 300 mg given every other week)	16	Instructed to apply adequate delgocitinib and undergo observation	98

*Eos*, Eosinophil; *LDH*, lactate dehydrogenase; *M*, male; *NA*, not applicable; *TARC*, thymus and activation-regulated chemokine.

The blood test results are the most recent values before the start of dupilumab treatment. The case 1 and 2 values were obtained 442 and 261 days before the start of dupilumab therapy, respectively.

## CASE 1

**Fig 1:**
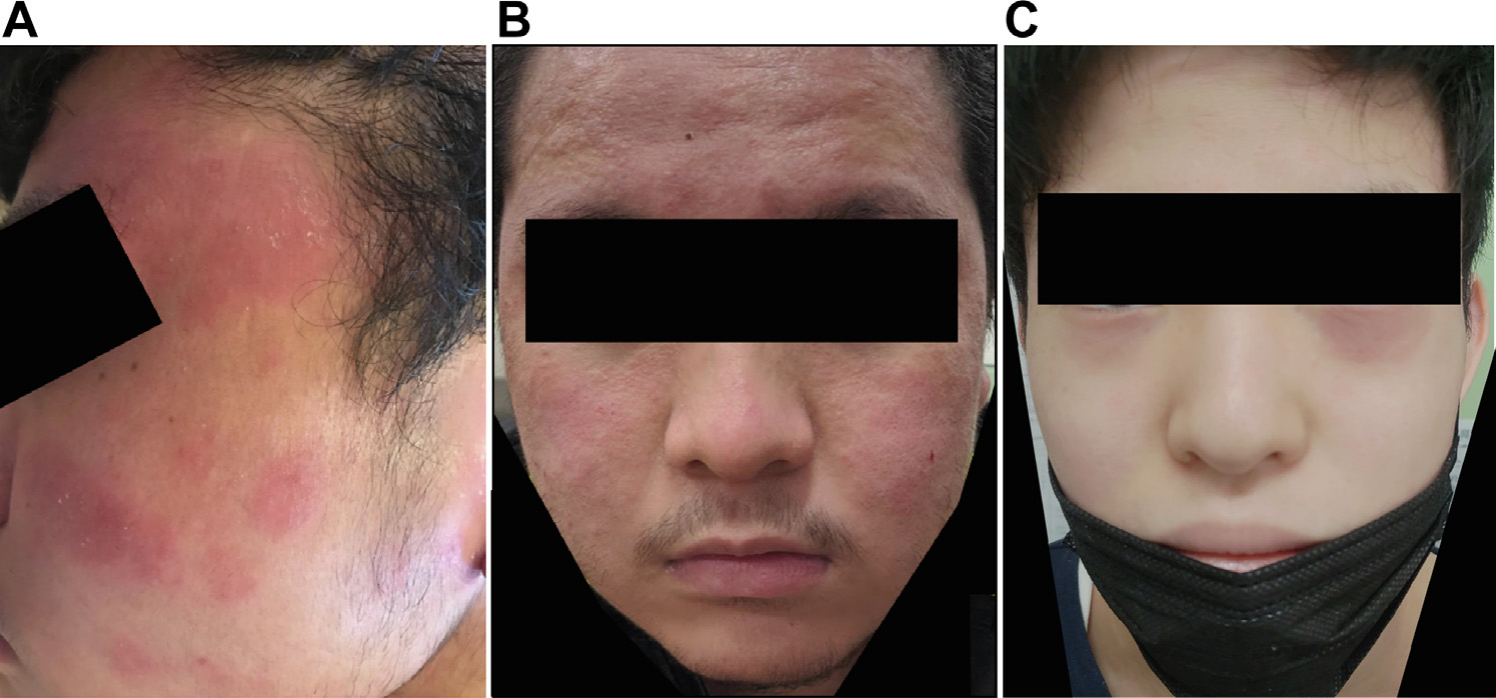
**A,** Case 1: a 16-year-old patient’s clinical picture of FE 2 months after starting dupilumab therapy. **B,** Case 2: a 15-year-old patient’s clinical picture of FE 2 months after starting dupilumab therapy. **C,** Case 3: a 17-year-old patient’s clinical picture of erythema localized to the lower eyelid 16 days after starting dupilumab therapy.

The patient was a 16-year-old boy with an EASI score of 16.1 and slight facial erythema (FE) at the initiation of dupilumab treatment. One month after the patient started receiving dupilumab, the eczema on his trunk and extremities alleviated, whereas his FE gradually worsened. The patient was followed up with instructions for application of topical corticosteroids (TCSs) class II. His FE became prominent at 3 months (Fig 1, *A*); it was barely itchy and did not cause a burning sensation. We instructed him to apply ketoconazole in addition to the TCS, which was not effective and was discontinued after about 2 weeks. Eventually, treatment with the TCS was continued, and improvement was observed at 4 months. As the patient's FE subsided, his treatment was changed from the TCS to delgocitinib, and the FE completely resolved at 5 months after the start of dupilumab treatment.

## Case 2

The patient was a 15-year-old boy with an EASI score of 10.5. He started receiving dupilumab because of eczema on his head, neck, trunk, and extremities that did not improve with the class Ⅱ TCS. One month after the patient started dupilumab treatment, his eczema had almost disappeared, and the facial ointment was switched from the TCS to topical calcineurin inhibitors (TCIs), which had been used previously without any adverse events. Two months after the start of dupilumab treatment, erythema without the itchiness and burning sensation appeared on his face (Fig 1, *B*); however, his trunk and extremities remained in good condition. Despite changing the ointment back to a TCS and instructing him to apply it thoroughly, the patient's FE persisted. Eventually his FE began to improve at 3 months after he started dupilumab and disappeared at 6 months.

## Case 3

The patient was a 17-year-old boy with an EASI score of 25.9. The patient continued to use delgocitinib ointment throughout his body after starting treatment with dupilumab, but 16 days after the start of dupilumab, he developed erythema localized to the lower eyelid without the itchiness and burning sensation (Fig 1, *C*). Delgocitinib ointment was applied firmly to his FE, which then improved at 2 months and completely disappeared at 3 months after he started receiving dupilumab.

This study is the first report of DFR in Japanese adolescents with AD. The incidence of DFR in all ages has been reported to be 9.9%,^2^ whereas the incidence of DFR in Japanese adolescents in this study was 23.1%, which was relatively high. The mean time to onset of DFR has been previously reported to be 11 weeks after initiation of dupilumab treatment,^3^ and the onset in the 3 patients in this study occurred at 2 to 12 weeks. The appearance of DFR should be monitored for about 1 year after initiation of dupilumab.

De Wijs et al^5^ reported that histopathology of DFR did not show typical acute-phase AD findings, suggesting a possible hypersensitivity reaction to dupilumab. Other possible causes include contact dermatitis, rosacea, steroid withdrawal, and Malassezia hypersensitivity.^6^ In the second case, the TCI could be a cause of FE. However, FE without itching or burning sensation was more consistent with the features of DFR^5^ than with those of a TCI-induced reaction; furthermore, there was no improvement with the discontinuation of TCI. Biopsy and patch test results have been reported as possible diagnostic clues,^5^^,^^7^ but these tests were not performed in our cases, as the patients showed improvements before testing. With respect to cytokine levels at the time of DFR emergence, we were able to measure IL-4 only in case 1, which showed a slightly elevated value of 3.6 pg/mL. Therefore, the association between IL-4 and DFR was not known in this study. As DFR cases accumulate and cytokine profiles advance, the mechanisms of DFR may be elucidated. In addition, measurement of neuropeptides may reveal the relationship between neurogenic inflammation and DFR.^8^

We treated the DFR with a TCS and antifungal ointment in case 1, a TCS and TCI in case 2, and delgocitinib ointment in case 3. Different to what was previously reported,^5^, ^9^ none of these treatments were clearly effective, but the DFR subsided without discontinuation of dupilumab within 3 months after onset. However, considering that dupilumab was discontinued in approximately 10% of previously reported cases owing to severe or prolonged DFR,^3^ careful follow-up is necessary. Because of the small number of patients in this study, further data on adolescent patients with AD treated with dupilumab are needed. In conclusion, DFR was observed in 3 Japanese adolescents with refractory AD, all of whom improved without prolongation.
